# Gut Region-Specific Interleukin 1β Induction in Different Myenteric Neuronal Subpopulations of Type 1 Diabetic Rats

**DOI:** 10.3390/ijms24065804

**Published:** 2023-03-18

**Authors:** Afnan AL Doghmi, Bence Pál Barta, Abigél Egyed-Kolumbán, Benita Onhausz, Szilvia Kiss, János Balázs, Zita Szalai, Mária Bagyánszki, Nikolett Bódi

**Affiliations:** Department of Physiology, Anatomy and Neuroscience, University of Szeged, 6726 Szeged, Hungary

**Keywords:** interleukin 1β enteric nervous system, myenteric neurons, neuronal nitric oxide synthase, calcitonin gene-related peptide, type 1 diabetes, hyperglycemia, insulin, gut regions

## Abstract

Interleukin 1β (IL1β) is a pro-inflammatory cytokine that may play a crucial role in enteric neuroinflammation in type 1 diabetes. Therefore, our goal is to evaluate the effects of chronic hyperglycemia and insulin treatment on IL1β immunoreactivity in myenteric neurons and their different subpopulations along the duodenum–ileum–colon axis. Fluorescent immunohistochemistry was used to count IL1β expressing neurons as well as the neuronal nitric oxide synthase (nNOS)- and calcitonin gene-related peptide (CGRP)-immunoreactive myenteric neurons within this group. Tissue IL1β level was measured by ELISA in muscle/myenteric plexus-containing homogenates. IL1β mRNA was detected by RNAscope in different intestinal layers. The proportion of IL1β-immunoreactive myenteric neurons was significantly higher in the colon than in the small intestine of controls. In diabetics, this proportion significantly increased in all gut segments, which was prevented by insulin treatment. The proportion of IL1β-nNOS-immunoreactive neurons only increased in the diabetic colon, while the proportion of IL1β-CGRP-immunoreactive neurons only increased in the diabetic ileum. Elevated IL1β levels were also confirmed in tissue homogenates. IL1β mRNA induction was detected in the myenteric ganglia, smooth muscle and intestinal mucosa of diabetics. These findings support that diabetes-related IL1β induction is specific for the different myenteric neuronal subpopulations, which may contribute to diabetic motility disturbances.

## 1. Introduction

Inflammatory cytokines, such as tumor necrosis factor alpha (TNFα) or different interleukins (ILs), trigger chronic low-grade inflammation that is linked to the development and progression of type 1 diabetes [1]. Due to the regionality of the intestinal structure and function, the regional cellular environment that accompanies diabetic gastroenteropathy has received special attention over the last decade [2,3,4]. Among others, the enteric neuronal microenvironment plays a pivotal role in the disturbed regulation of gut motility in diabetic patients [5,6,7]. However, the microbial environment of the intestinal epithelium, mucosal, submucosal and muscular milieu is also essential to the cellular crosstalk vertically in the gut wall [8].

Cytokines have global impacts on intestinal health and disease [9,10,11,12]. It has recently been shown that chronic hyperglycemia has regionally different effects on TNFα expression in distinct gut segments [13]. Moreover, the expression of TNFα receptors is also differently altered in the different segments; TNF receptor 2 is more affected than TNF receptor 1 in the duodenal myenteric ganglia of type 1 diabetic rats [14].

The IL1-family of cytokines consists of 11 members and is produced primarily by the vast majority of immune cells in response to microbial stimuli or other cytokines [15]. Among them, IL1 has two distinct isoforms, IL1α and IL1β, with only 26% homology between them. Pro-IL1β is functionally inactive; its cleavage is mediated by caspase-1 in inflammasomes, and then as a bioactive pro-inflammatory mediator it amplifies inflammatory cascades and regulates innate immunity [15,16,17]. IL1 has roles both in autoimmune and autoinflammatory diseases [18,19,20].

IL1β promotes both systemic and tissue inflammation in diabetes, including β-cell apoptosis of pancreatic islets and diabetic cardiovascular complications related to increased IL1β expression in the aorta endothelium, heart and retinal vessels of diabetic rats [21,22]. IL1β and other pro-inflammatory cytokines are also elevated in inflammatory bowel disease and necrotizing enterocolitis, inducing intestinal inflammation through the disruption of the epithelial junctional barrier [23]. IL1β also serves as a key factor in the development and maintenance of neuropathic pain in different chronic states by mediating neuro-immune interactions in the spinal cord and brain [24]. However, so far only a few studies have focused on the role that IL1β plays in the enteric nervous system [25,26,27].

Diabetic enteric neuropathy has been studied extensively [28,29], especially as the vast majority of diabetic patients suffer from gastrointestinal symptoms such as nausea, vomiting, diarrhea or constipation. In the background of these diabetic motility disturbances, the nitrergic myenteric subpopulation is particularly affected [5,30,31]. Nitrergic myenteric neurons (containing neuronal nitric oxide synthase, nNOS) represent a robust neuronal population in the myenteric plexus [32,33]. Most of these cells are inhibitory interneurons or motor neurons that are responsible for the descending inhibition of gut motility. However, nNOS neurons also have an important role in the inflammatory responses of enteric neurons in traumatic nerve injury, inflammatory bowel diseases or ischaemic damage [34,35,36]. On the other hand, intrinsic primary afferent neurons (IPANs), as transducers, are responsible for the detection of mechanical and chemical stimuli and for the initiation of enteric reflexes in the gut wall [37], therefore, their involvement is also crucial in peristalsis and other gut functions during diabetes. IPANs are characterized by Dogiel type II morphology and many of them contain calcitonin gene-related peptide (CGRP) [37,38] which is affected in diabetes [39].

Different subpopulations of myenteric neurons respond differently to diabetic damage [31,40,41]. Therefore, we presumed that inflammatory responses of these neuronal populations may also contain differences. The primary goal of this study was to evaluate the effects of chronic hyperglycemia and immediate insulin treatment on the proportion of IL1β-immunoreactive (IR) myenteric neurons along the duodenum–ileum–colon axis. Moreover, we aimed to reveal whether a neuronal population-specific IL1β immunoreactivity exists in the nNOS-IR and CGRP-IR subpopulations in type 1 diabetic rats.

## 2. Results

### 2.1. Weight and Glycemic Characteristics of Experimental Rats

The weight and blood glucose concentration of the diabetic, insulin-treated diabetic and control rats were monitored during the 10-week experiment and are summarized in Table 1.

Diabetic rats were characterized by a long-lasting chronic hyperglycemia, their average blood glucose concentration was 27.05 ± 1.12 mmol/L, which was more than four times higher than that of the controls (5.81 ± 0.1 mmol/L). Immediate insulin treatment inhibited extremely high glucose levels, however, these were still higher than in the control group (12.57 ± 1.19 mmol/L). All the experimental rats gained weight during the 10-week experimental period, although the final body weight of diabetic animals was significantly lower compared to the insulin-treated diabetic (*p* < 0.001) and control (*p* < 0.0001) rats.

### 2.2. Gut Region-Specific Presence and Diabetic Induction of IL1β-Immunoreactive Myenteric Neurons

**Figure 1:**
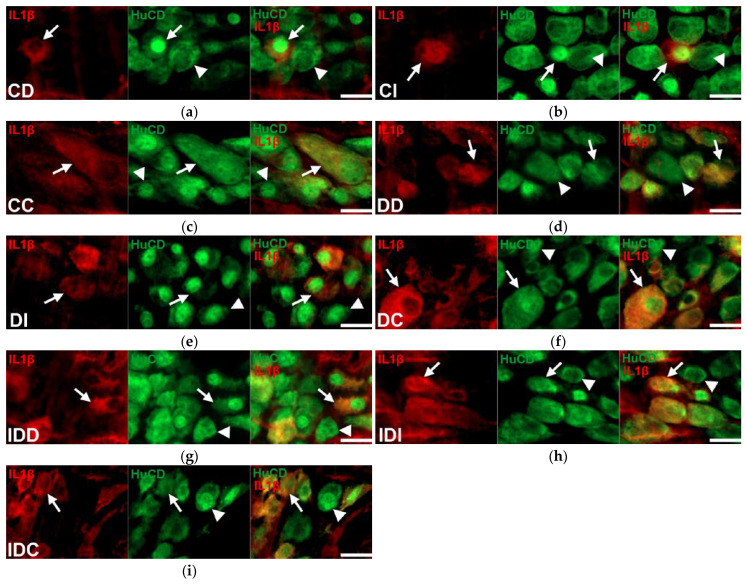
Representative fluorescent micrographs of whole-mount preparations of myenteric ganglia from the duodenum, ileum and colon of control, diabetic, and insulin-treated diabetic rats after IL1β-HuC/HuD double-labelling immunohistochemistry. HuC/HuD as a pan-neuronal marker was applied to label myenteric neurons. CD—control duodenum (**a**), CI—control ileum (**b**), CC—control colon (**c**), DD—diabetic duodenum (**d**), DI—diabetic ileum (**e**), DC—diabetic colon (**f**), IDD—insulin-treated diabetic duodenum (**g**), IDI—insulin-treated diabetic ileum (**h**), IDC—insulin-treated diabetic colon (**i**); arrows—IL1β-immunoreactive myenteric neurons, arrowheads—myenteric neurons. Scale bars: 20 μm.

Double-labelling fluorescent immunohistochemistry revealed regional differences in IL1β immunoreactivity in myenteric neurons along the intestinal tract in control animals (Figure 1).

**Figure 2:**
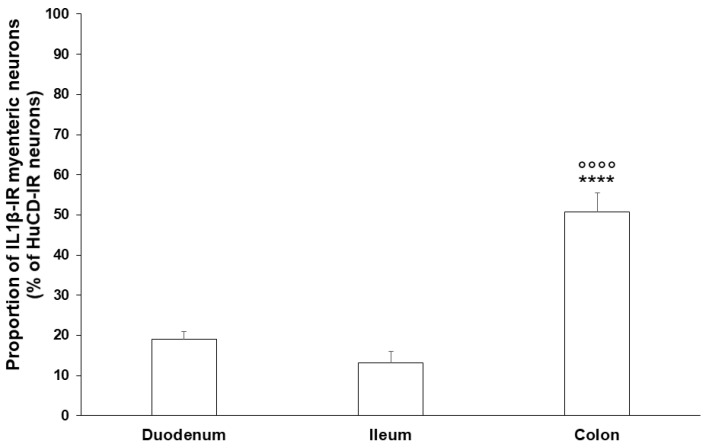
Proportion of IL1β-immunoreactive myenteric neurons in the duodenum, ileum, and colon of control rats. The proportion of IL1β-immunoreactive myenteric neurons was significantly higher in the colon compared to the ileum and duodenum. Data are expressed as mean ± SEM. **** *p* < 0.0001 (relative to control duodenum); ^oooo^ *p* < 0.0001 (between control ileum and colon).

The proportion of IL1β-IR myenteric neurons compared to the total myenteric neuronal number was lower in the small intestine (duodenum: 18.97 ± 2.05%, ileum: 13.11 ± 2.93%), than in the colonic ganglia, where it was significantly, more than 50%, higher (50.7 ± 4.75%, *p* < 0.0001) (Figure 2).

**Figure 3:**
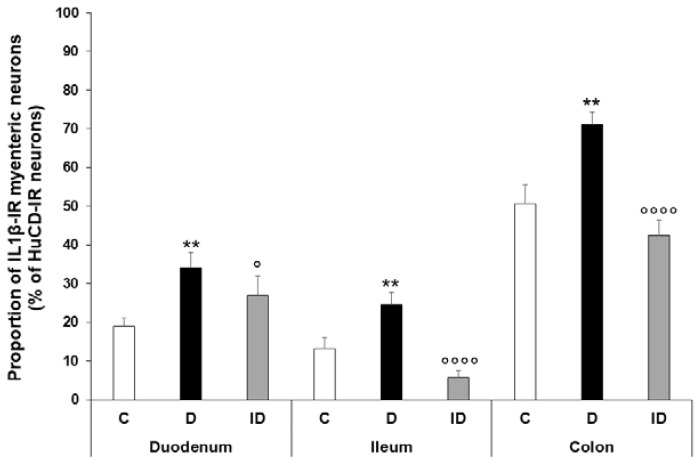
Proportion of IL1β-immunoreactive myenteric neurons of the duodenum, ileum, and colon of control, diabetic and insulin-treated diabetic rats. In the diabetics, the proportion of IL1β-immunoreactive myenteric neurons was significantly increased in all gut segments, which was prevented by immediate insulin treatment. Data are expressed as mean ± SEM. ** *p* < 0.01 (relative to controls); ^o^ *p* < 0.05, ^oooo^ *p* < 0.0001 (between diabetics and insulin-treated diabetics). C—controls, D—diabetics, ID—insulin-treated diabetics.

In diabetic rats, the IL1β-IR myenteric neuronal proportion was significantly increased in all investigated gut segments (*p* < 0.01); it was almost doubled in the duodenum and ileum (34.04 ± 4.06% and 24.62 ± 3.11%, respectively), while it increased by more than 20% in the colon (71.14 ± 3.29%) (Figure 3). In the insulin-treated group, the proportion of IL1β-IR myenteric neurons was not altered significantly compared to the control levels (Figure 3).

### 2.3. Region-Dependent Increase of IL1β Immunoreactivity in nNOS-Immunoreactive Myenteric Neurons of Diabetic Rats

**Figure 4:**
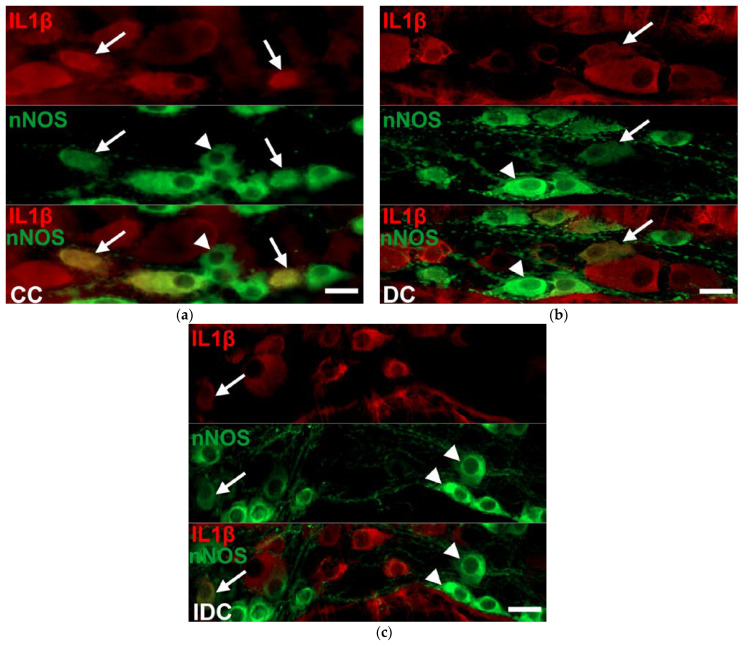
Representative fluorescent micrographs of whole-mount preparations of myenteric ganglia from the colon of control (**a**), diabetic (**b**), and insulin-treated diabetic (**c**) rats after IL1β-nNOS double-labelling immunohistochemistry. CC—control colon, DC—diabetic colon, IDC—insulin-treated diabetic colon; arrows—IL1β-nNOS-immunoreactive myenteric neurons, arrowheads—nNOS-immunoreactive neurons. Scale bars: 20 μm.

**Figure 5:**
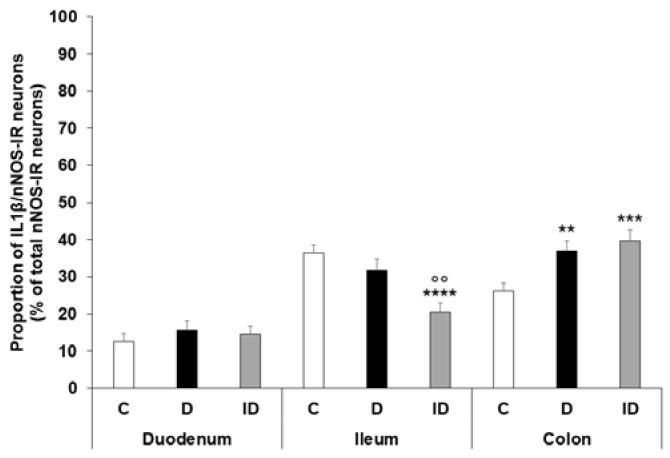
Proportion of IL1β-nNOS-immunoreactive neurons related to the total number of nNOS-immunoreactive neurons in the myenteric ganglia of the duodenum, ileum, and colon of control, diabetic and insulin-treated diabetic rats. The proportion of IL1β-nNOS-immunoreactive neurons significantly increased only in the colon of diabetics relative to controls. Data are expressed as mean ± SEM. ** *p* < 0.01, *** *p* < 0.001, **** *p* < 0.0001 (relative to controls); ^oo^ *p* < 0.01 (between diabetics and insulin-treated diabetics). C—controls, D—diabetics, ID—insulin-treated diabetics.

IL1β and nNOS double-labelling immunofluorescence was applied to label IL1β-containing nitrergic neurons in the myenteric ganglia (Figure 4). The proportion of IL1β-nNOS-IR cells per total nNOS-IR myenteric neurons was the lowest in the duodenum (12.64 ± 2.03%) and significantly higher in the distal segments (ileum: 36.36 ± 2.18; colon: 26.21 ± 2.13; *p* < 0.0001) of the control animals. In the diabetics, the colon was the only segment where this proportion was significantly increased (36.94 ± 2.76% vs. 26.21 ± 2.13%, *p* < 0.01), while it remained unchanged in the duodenal and ileal ganglia (Figure 5). The immediate insulin treatment did not protect against the diabetic induction of IL1β expression in the nitrergic neuronal population of colonic ganglia, and decreased the IL1β-nNOS-IR neuronal proportion in the ileum (Figure 5).

### 2.4. Region-Dependent Increase of IL1β Immunoreactivity in CGRP-IR Myenteric Neurons of Diabetic Rats

**Figure 6:**
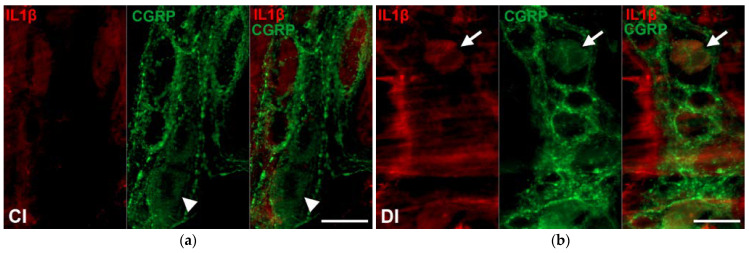
Representative fluorescent micrographs of whole-mount preparations of myenteric ganglia from the ileum of control (**a**) and diabetic (**b**) rats after IL1β-CGRP double-labelling immunohistochemistry. CI—control ileum, DI—diabetic ileum; arrows—IL1β-CGRP-immunoreactive myenteric neurons, arrowheads—CGRP-immunoreactive neurons. Scale bars: 20 μm.

Myenteric neurons immunoreactivity for both IL1β and CGRP were made visible by double-labelling fluorescent immunohistochemistry on whole-mount preparations (Figure 6).

**Figure 7:**
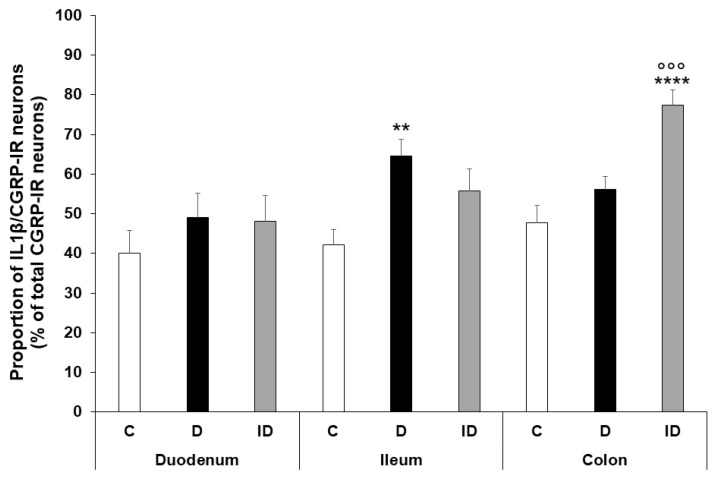
Proportion of IL1β-CGRP-immunoreactive neurons related to the total number of CGRP-immunoreactive neurons in the myenteric ganglia of the duodenum, ileum, and colon of control, diabetic and insulin-treated diabetic rats. The proportion of IL1β-CGRP-immunoreactive neurons significantly increased only in the ileum of diabetics relative to controls. Data are expressed as mean ± SEM. ** *p* < 0.01, **** *p* < 0.0001 (relative to controls); ^ooo^ *p* < 0.001 (between diabetics and insulin-treated diabetics). C—controls, D—diabetics, ID—insulin-treated diabetics.

The proportion of IL1β-CGRP-IR neurons per total CGRP-IR myenteric neurons was between 40–50% in all intestinal regions of control animals. This proportion was only significantly altered in the ileum of diabetics, where it increased compared to the controls (64.56 ± 4.17 vs. 42.13 ± 3.8, *p* < 0.01) (Figure 7). In the insulin-treated group, an increased IL1β-CGRP neuronal proportion was observed in the colonic ganglia compared to both controls and diabetics (Figure 7).

### 2.5. Increased Tissue Level of IL1β in Colonic Muscle/Myenteric Plexus Homogenates of Diabetics

**Figure 8:**
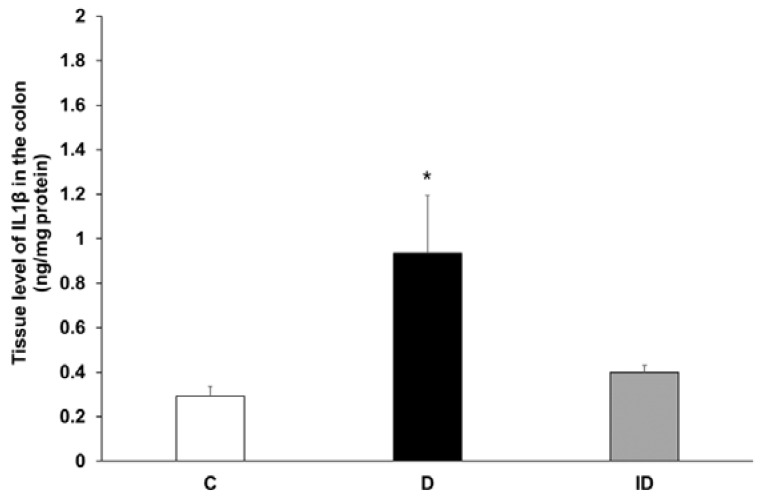
Tissue level of IL1β in intestinal smooth muscle layer homogenates including the myenteric plexus from the colon of control, diabetic and insulin-treated diabetic rats. The IL1β tissue level was significantly increased in diabetics relative to controls, which was prevented by insulin treatment. Data are expressed as means ± SEM. * *p* < 0.05 (relative to controls). C—controls, D—diabetics, ID—insulin-treated diabetics.

The proportion of the IL1β-IR myenteric neurons was highest in control condition in the colon, hence the IL1β concentration was measured here in tissue homogenates that contained the intestinal smooth muscle and the myenteric plexus. The IL1β tissue level was 0.29 ± 0.04 ng/mg protein in control samples, while it was significantly higher in diabetic homogenates (0.94 ± 0.26 ng/mg, *p* < 0.05) and was restored to the control level in insulin-treated diabetic samples (0.4 ± 0.03 ng/mg) (Figure 8).

### 2.6. Quantitative Evaluation of IL1β mRNA Level

**Figure 9:**
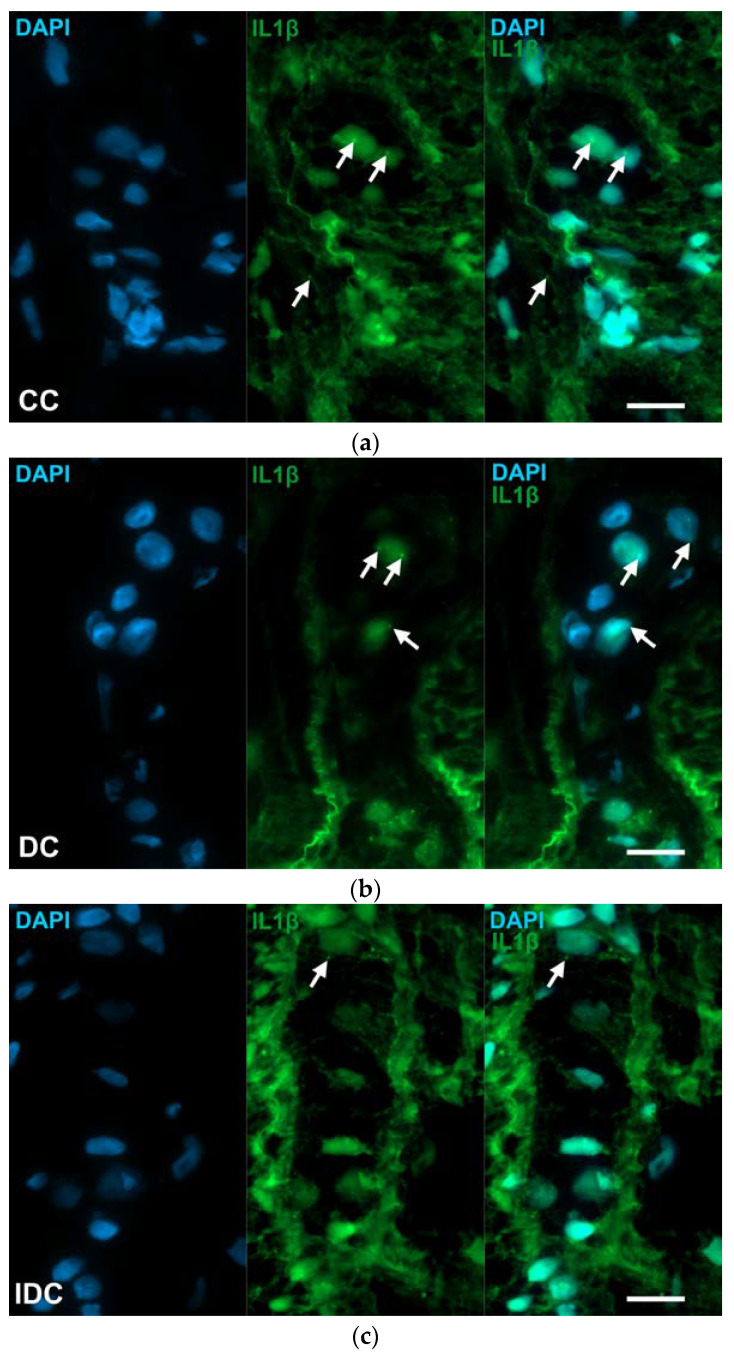
Representative micrographs of cryosections of myenteric ganglia from the colon of control (**a**), diabetic (**b**) and insulin-treated diabetic (**c**) rats after IL1β RNAscope. IL1β mRNA appear as green punctate dots (arrows), nuclei were counterstained with DAPI (blue). CC—control colon, DC—diabetic colon, IDC—insulin-treated diabetic colon. Scale bars: 20 μm.

**Figure 10:**
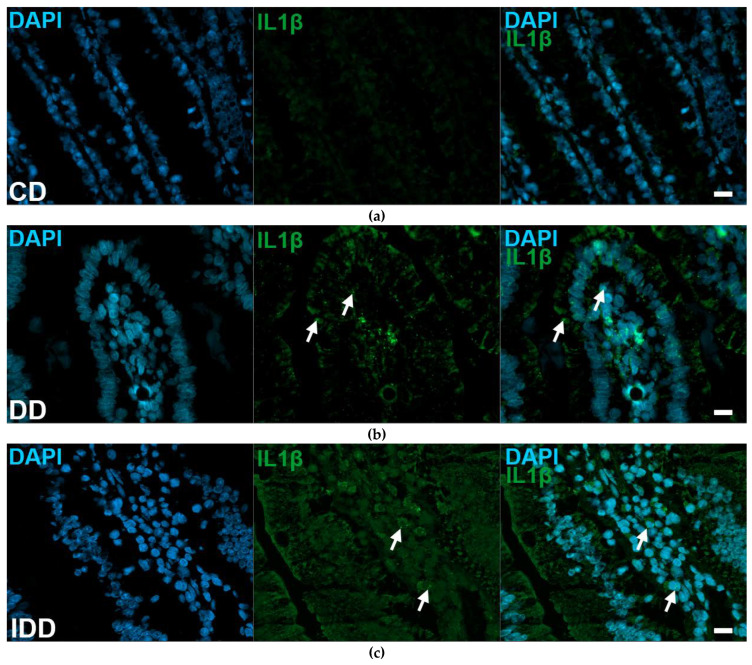
Representative micrographs of cryosections of mucosa from the duodenum of control (**a**), diabetic (**b**) and insulin-treated diabetic (**c**) rats after IL1β RNAscope. IL1β mRNA appear as green punctate dots (arrows), nuclei were counterstained with DAPI (blue). CD—control duodenum, DD—diabetic duodenum, IDD—insulin-treated diabetic duodenum. Scale bars: 20 μm.

The IL1β mRNA expression detected by RNAscope on cryosections in controls varied depending on the investigated gut region and intestinal layer (Figure 9 and Figure 10).

The number of punctate dots labelling IL1β mRNA was lowest in the intestinal smooth muscle layer in all gut segments of control rats, while it was highest in the myenteric ganglia of the duodenum and colon (3400 ± 429 and 3361 ± 279.5, respectively). In the ileum, the highest value was measured in mucosa (4558 ± 486.7), which was roughly double that of the duodenum and colon (Table 2).

**Figure 11:**
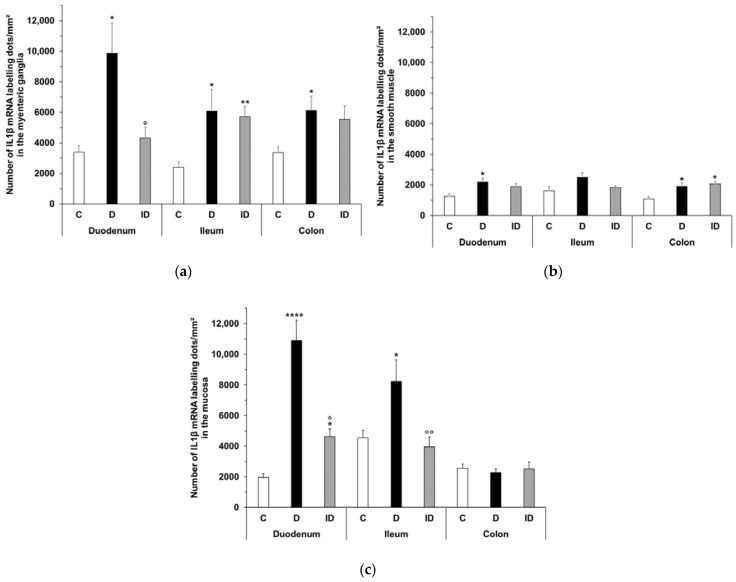
Expression of IL1β mRNA in the myenteric ganglia (**a**), smooth muscle (**b**), and intestinal mucosa (**c**) of the duodenum, ileum, and colon of control, diabetic and insulin-treated diabetic rats. Data are expressed as mean ± SEM. * *p* < 0.05, ** *p* < 0.01, **** *p* < 0.0001 (relative to controls); ^o^ *p* < 0.05, ^oo^ *p* < 0.01 (between diabetics and insulin-treated diabetics). C—controls, D—diabetics, ID—insulin-treated diabetics.

In the myenteric ganglia of diabetic rats, the number of IL1β mRNA labelling dots was significantly increased in all gut segments (*p* < 0.05); the greatest increase was observed in the duodenum where the dot density was almost tripled (Figure 11a). In insulin-treated diabetic rats, in the same gut region, the number of dots remained at control level (Figure 11a). In diabetics, the number of IL1β mRNA dots increased by a lesser extent in intestinal smooth muscle as well, and this change was significant in the duodenum and colon (*p* < 0.05; Figure 11b).

In the mucosa of diabetics, IL1β mRNA expression varied differently in different gut segments. A robust, more than five times increase in dot number was observed in the duodenum (*p* < 0.0001), and almost double in the ileum (*p* < 0.05), however, no change was detected in the colonic mucosa (Figure 11c). Insulin treatment completely prevented the diabetic alterations in the ileum and partially in the duodenum (Figure 11c).

## 3. Discussion

In the present study, a distinct IL1β induction was demonstrated in myenteric neurons in all gut segments of type 1 diabetic rats. Moreover, a strict regionality was revealed in IL1β immunoreactivity in different myenteric subpopulations.

Although IL1β is primarily produced by macrophages, other cell types, such as epithelial and endothelial cells, smooth muscle cells, neurons and glial cells, can also express it [20,24,42,43] and several factors can influence its release [16]. In control animals, a great difference in the proportion of IL1β-IR myenteric neurons between the small and large intestine was observed. The high baseline level of IL1β may be explained by an excessive pro-oxidant environment in the colon [44,45]. Similarly, other important members of the host defense and innate immunity, such as TNFα and TLR4, display higher basal distribution in the colon than in the small intestine [13,46,47].

A general induction of IL1β expression was revealed in myenteric neurons of diabetic rats, and this induction was prevented by immediate insulin treatment. Furthermore, our observation was also confirmed in the homogenates of intestinal smooth muscle layers including the myenteric plexus prepared from the gut wall. These results reflect that the entire enteric nervous system reacts to the long-term hyperglycemic state and suggest a role for IL1β in enteric neuroinflammation. A high glucose concentration itself can activate IL1β precursors in different cells [48,49], which then stimulate nuclear factor kappa B activation [50], a key element of many pro-inflammatory signaling pathways. Pro-inflammatory cytokines are able to facilitate neurite growth via the upregulation of neurotrophic factors in annulus cells [51,52], cortical neural precursors [53] and myenteric neurons [25].

When investigating the nNOS-IR myenteric population in healthy controls, a higher proportion of IL1β-containing nitrergic neurons was observed in distal gut segments, similarly to the total number of IL1β-IR myenteric neurons. In contrast, IL1β-containing CGRP-IR myenteric neurons exhibited an equal distribution along the duodenum–ileum–colon axis and their proportion was relatively high, 40–50%, among the total number of CGRP-IR cells. This discrepancy refers to a neuronal population-specific IL1β expression in itself.

In diabetics, the proportion of IL1β-containing nitrergic neurons increased only in the colonic segment, while it did not change elsewhere. Former studies [2,5] demonstrated that the nitrergic myenteric system exhibits a regional perturbation in the diabetic state, with the density of these inhibitory neurons decreasing in all intestinal segments, while this was not accompanied with neuronal loss in the duodenal ganglia. Considering these findings, it is interesting to note that, despite the nitrergic loss in the diabetic colon, the proportion of IL1β-containing nNOS-IR neurons still increased. Other studies have shown that IL1β is involved in the stimulation of nuclear factor kB-mediated inducible NOS gene expression [54,55]. It has also been demonstrated that IL1β specifically activates different neuronal and glial cell populations in the myenteric and submucous plexuses of the guinea pig ileum and colon [26]. The majority of activated myenteric neurons were NOS-IR, while most activated submucous neurons expressed vasoactive intestinal polypeptide in both gut segments [26].

Additionally, the proportion of IL1β-containing CGRP-IR neurons was elevated only in the ileum and remained unchanged in the duodenal and colonic ganglia of diabetics. As IPANs are essential to sense luminal stimuli, the alterations in microbial composition in diabetes may serve as a possible explanation of our results. Namely, a large-scale microbial rearrangement and robust *Klebsiella* invasion [4], together with a notable endogenous heme oxygenase defense system induction, has been detected in the ileum [2]. Several studies have demonstrated that CGRP-like immunoreactivity was decreased in both enteric plexuses of the diabetic rat ileum and colon [39,56,57], while others have observed an increased number of CGRP-containing neurons in the pig small intestine [58]. Hou et al. showed that IL1β can directly induce the release of CGRP from sensory neurons in a concentration-dependent manner [59]. As CGRP can modulate immune cell function, the role of neuropeptide–cytokine interactions draws attention to the importance of further investigations in diabetic enteric neuropathy [39].

At the transcriptional level, IL1β mRNA induction was shown to occur in the myenteric ganglia in all investigated gut segments of diabetic rats and the IL1β mRNA level was also increased in intestinal smooth muscle. The number of IL1β mRNA-labelling dots clearly indicates that the smooth muscle of the gut wall is also a source of cytokines, although it is less affected in diabetes than the ganglia. Enhanced IL1β mRNA expression was shown in the colonic muscle of rats with colitis [60]. Furthermore, IL1β can induce IL6 mRNA and protein expression in cultured muscle cells of the rat jejunum [61]. The mucosal IL1β mRNA level was robustly increased in the small intestine, especially in the duodenum of diabetics. Diabetic alterations of mucosa-associated gut microbiota [62] may induce mucosal IL1β production [63] and contribute to an increase of intestinal permeability [9,23].

In summary, our results underline the induction of IL1β in myenteric neurons, however, the different myenteric neuronal populations are affected in a region-dependent manner. The nNOS-IR myenteric neurons were most affected in the colon, while CGRP-IR neurons were most susceptible in the ileum of diabetic rats. Therefore, IL1β not only determines the enteric inflammatory environment during type 1 diabetes, but may also be involved in diabetes-related alterations of enteric neurochemical phenotype, and may participate in population-specific activation of myenteric neurons leading to diabetic motility disturbances. These results may contribute to the wider use of IL1-based therapies introduced in recent years in autoinflammatory diseases [18,19,64].

## 4. Materials and Methods

### 4.1. Animal Model

Adult male Wistar rats (Toxi-Coop Zrt., Balatonfüred, Hungary) weighing 200–250 g, kept on standard laboratory chow (Innovo Kft., Zsámbék, Hungary) and with free access to drinking water, were used in the experiments. The rats were divided randomly into three groups: streptozotocin (STZ)-induced diabetics (diabetics; n = 15), insulin-treated STZ-induced diabetics (insulin-treated diabetics; n = 14), and sex- and age-matched controls (n = 16). Hyperglycemia was induced by a single intraperitoneal injection of STZ (Sigma–Aldrich, Budapest, Hungary) at 60 mg/kg [5,13,65]. The animals were considered diabetic if the non-fasting blood glucose concentration was higher than 18 mmol/L. From this time on, the insulin-treated group of hyperglycemic rats received a subcutaneous insulin injection (Humulin M3, Eli Lilly Nederland, Utrecht, The Netherlands) each morning (2 IU) and afternoon (3 IU). Equivalent volumes of saline were administered subcutaneously to the diabetic and control groups. The blood glucose levels and animal weights were measured weekly. Those diabetic animals that recovered spontaneously, or whose glucose levels decreased less than 18 mmol/L during the 10-week period, were excluded from the study. In all procedures involving experimental animals, the principles of the National Institutes of Health (Bethesda, MD, USA) guidelines and the EU directive 2010/63/EU for the protection of animals used for scientific purposes were strictly followed, and all of the experiments were approved by the National Scientific Ethical Committee on Animal Experimentation (National Competent Authority), with the license number XX./1636/2019.

### 4.2. Tissue Handling

Ten weeks after the onset of hyperglycemia, the animals were sacrificed by cervical dislocation under chloral hydrate anesthesia (375 mg/kg i.p.). The gut segments of diabetic, insulin-treated diabetic and control rats were dissected and rinsed in 0.05 M phosphate buffer (PB; pH 7.4). Tissue samples were taken from the duodenum (1 cm distal to the pylorus), the ileum (1 cm proximal to the ileo-cecal junction), and the proximal colon, and processed for double-labelling fluorescent immunohistochemistry, enzyme-linked immunosorbent assay (ELISA), and RNAscope multiplex fluorescent V2 assay (RNAscope). For fluorescent immunohistochemistry, the intestinal segments were cut along the mesentery, pinched flat, and fixed overnight at 4 °C in 4% formaldehyde solution buffered with 0.1 M PB (pH 7.4). The samples were then washed, the mucosa, submucosa, and circular smooth muscle were removed, and whole-mount preparations with the myenteric plexus adhering to the longitudinal smooth muscle were prepared. For the ELISA, the 3-cm-long gut segments were cut along the mesentery and pinched flat. After removing both the mucosa and submucosa, the layers of intestinal smooth muscle including the myenteric plexus were snap-frozen in liquid nitrogen and stored at −80 °C until use. For the RNAscope, small pieces (2–3 mm) of the gut segments were fresh-frozen in liquid nitrogen, embedded in cryomatrix medium (O.C.T.^TM^, Tissue-Tek, Sakura, The Netherland) and stored at −80 °C until use.

### 4.3. Fluorescent Immunohistochemistry

For immunofluorescence studies, IL1β-HuC/HuD, IL1β-nNOS or IL1β-CGRP double-labelling immunohistochemistry were performed on whole-mounts derived from different gut segments. Briefly, after blocking in tris(hydroxymethyl)aminomethane-buffered saline (TBS) containing 1% bovine serum albumin and 10% normal goat serum, the whole-mount preparations were incubated overnight with anti-IL1β (mouse monoclonal IgG; sc-52012, Santa Cruz Biotechnology, Dallas, TX, USA; final dilution 1:50) and pan-neuronal anti-HuC/HuD (rabbit monoclonal IgG; ab184267, Abcam, Cambridge, UK; final dilution 1:200) or anti-nNOS (rabbit polyclonal IgG; Cat. No. 160870, Cayman Chemical, Ann Arbor, MI, USA; final dilution 1:200) or anti-CGRP (rabbit polyclonal IgG; PC205L, Calbiochem, Merck, Germany; final dilution 1:100) primary antibodies at 4 °C. After washing in TBS with 0.025% Triton X-100, sections were incubated with anti-mouse Cy^TM^3 (Jackson ImmunoResearch Laboratories, West Grove, PA, USA; final dilution 1:200) and anti-rabbit Alexa Fluor 488 (A11008, Invitrogen, Thermo Fisher Scientific, Waltham, MA, USA; final dilution 1:200) secondary antibodies for 1 h at room temperature. Negative controls were performed by omitting the primary antibodies when no immunoreactivity was observed. Whole-mounts were mounted on slides in Fluoromount^TM^ Aqueous Mounting Medium (Sigma–Aldrich, Budapest, Hungary), and observed and photographed with a Zeiss Imager Z.2 fluorescent microscope equipped with an Axiocam 506 mono camera (Zeiss, Germany, Jena). Fifty myenteric ganglia were taken from each intestinal segment from each experimental group, and the proportion of myenteric neurons that are immunoreactive for either IL1β, IL1β-nNOS or IL1β-CGRP were counted per ganglia.

### 4.4. Measurement of Tissue IL1β Concentration

Samples from the colon, including the intestinal smooth muscle layers with the myenteric plexus in between, were frozen in liquid nitrogen, crushed into powder in a mortar and homogenized in 500 µL homogenizing buffer (100 µL Protease Inhibitor Cocktail (Sigma–Aldrich, Budapest, Hungary) in 20 mL 0.05 M PB). Tissue homogenates were centrifuged at 5000 rpm for 20 min at 4 °C. The IL1β levels of the colon samples were determined by means of quantitative ELISA according to the manufacturer’s instructions (GA-E0128RT, GenAsia Biotech Co., Shanghai, China). Optical density was measured at 450 nm (Benchmark Microplate Reader; Bio-Rad, Budapest, Hungary). The tissue IL1β concentration was expressed as ng/mg protein.

### 4.5. Bradford Protein Micromethod for the Determination of Tissue Protein Content

A commercial protein assay kit was used for the determination of protein content in intestinal smooth muscle/myenteric plexus homogenates of colon. Bradford reagent was added to each sample. After mixing and following 10 min incubation, the samples were assayed spectrophotometrically at 595 nm. The protein level was expressed as mg protein/mL.

### 4.6. RNAscope Multiplex Fluorescent V2 Assay

To detect IL1β mRNA in different intestinal layers, the RNAscope Multiplex Fluorescent V2 Assay (Cat. No. 323100, Advanced Cell Diagnostics, Newark, CA, USA) was used according to the manufacturer’s instructions. Briefly, fresh-frozen cryosections (5 µm) were fixed in 4% formaldehyde and dehydrated in increasing ethanol concentrations. The cryosections were incubated in hydrogen peroxide, followed by protease IV treatment and then hybridized with a rat IL1β probe (Cat. No. 314011-C2, Advanced Cell Diagnostics, Newark, CA, USA; final dilution 1:50) for 2 h at 40 °C. After a three-step signal amplification and HRP signal development combined with fluorophore (Opal^TM^ 520 Reagent Pack, FP1487001KT, Akoya Biosciences, Menlo Park, CA, USA; final dilution 1:100, 30 min at 40 °C), tissues were counterstained with DAPI, mounted with Fluoromount^TM^ Aqueous Mounting Medium (Sigma–Aldrich, Budapest, Hungary) and photographed with a Zeiss Imager Z.2 fluorescent microscope equipped with an Axiocam 506 mono camera (Zeiss, Germany, Jena). Positive and negative control probes were also run to assess sample RNA quality and optimal permeabilization. Fifteen digital photographs of intestinal mucosa, smooth muscle and myenteric ganglia were taken from each gut segment and experimental group, and the number of punctate dots labelling IL1β mRNA was evaluated (dots per unit area).

### 4.7. Statistical Analysis

A statistical analysis was performed with Kruskal–Wallis test with a Dunn’s multiple comparisons test. All analyses were carried out with GraphPad Prism 6.0 (GraphPad Software, San Diego, CA, USA). A probability of *p* < 0.05 was set as the level of significance. All data are expressed as means ± SEM.

## Figures and Tables

**Table 1:** Weight and glycaemic characteristics of the experimental animals.

	Weight (g)		Blood Glucose Level (mmol/L)	
	Initial	Final	Initial	Final (Average)
Controls (n = 16)	206 ± 2.48	445.6 ± 13.58 ^c^	4.82 ± 0.29	5.81 ± 0.1
Diabetics (n = 15)	206.1 ± 2.57	317.7 ± 13.34 ^a,e^	5.47 ± 0.42	27.05 ± 1.12 ^c,e^
Insulin-treated diabetics (n = 14)	216.1 ± 5.37	432.1 ± 15.12 ^c,g^	5.21 ± 0.42	12.57 ± 1.19 ^b,d,f^

Data are expressed as mean ± SEM; ^a^ *p* < 0.05, ^b^ *p* < 0.001, ^c^ *p* < 0.0001 vs. initial; ^d^ *p* < 0.01, ^e^ *p* < 0.0001 vs. final controls; ^f^ *p* < 0.05, ^g^ *p* < 0.001 vs. final diabetics.

**Table 2:** Quantitative evaluation of IL1β mRNA labelling dots in different intestinal layers and gut segments of control rats.

	Duodenum (dots/mm^2^)	Ileum (dots/mm^2^)	Colon (dots/mm^2^)
Myenteric ganglia	3400 ± 429	2420 ± 486.7	3361 ± 279.5
Smooth muscle	1251 ± 181.5 ^b^	1629 ± 257.7	1080 ± 146 ^c^
Mucosa	1948 ± 281.4	4558 ± 486.7 ^a,e,f^	2549 ± 279.5 ^d,g^

Data are expressed as means ± SEM; ^a^ *p* < 0.05, ^b^ *p* < 0.001, ^c^ *p* < 0.0001 vs. myenteric ganglia in the same gut segment; ^d^ *p* <0.01, ^e^ *p* < 0.001 vs. smooth muscle in the same gut segment; ^f^ *p* < 0.001 vs. duodenum in the same intestinal layer; ^g^ *p* < 0.01 vs. ileum in the same intestinal layer.

## Data Availability

Dataset available from the corresponding author at bodi.nikolett@bio.u-szeged.hu.
